# Metagenomes and metagenome-assembled genomes from microbial communities in a biological nutrient removal plant operated at Hamptons Road Sanitation District (HRSD) with high and low dissolved oxygen conditions

**DOI:** 10.1128/mra.01492-25

**Published:** 2026-05-14

**Authors:** Blaise M. Enuh, Kevin S. Myers, Charles Bott, Stephanie Klaus, Kester McCullough, Lilian McIntosh, Natalie Beach, Michelle Young, Timothy J. Donohue, Daniel R. Noguera

**Affiliations:** 1 Great Lakes Bioenergy Research Center, University of Wisconsin-Madison 5228, Madison, Wisconsin, USA; 2 Wisconsin Energy Institute, University of Wisconsin-Madison 5228, Madison, Wisconsin, USA; 3 Hamptons Road Sanitation District, Virginia Beach, Virginia, USA; 4 Carollo Engineers, Westminster, Colorado, USA; 5 Department of Bacteriology, University of Wisconsin-Madison 5228, Madison, Wisconsin, USA; 6 Department of Civil and Environmental Engineering, University of Wisconsin-Madison 5228, Madison, Wisconsin, USA; Montana State University, Bozeman, Montana, USA

**Keywords:** metagenomics, microbial communities, wastewater treatment, dissolved oxygen

## Abstract

Aeration is a major cost at biological nutrient removal (BNR) plants. We report on microbial communities in a pilot-scale BNR system before and after a dissolved oxygen transition from 2.5 to 0.2 mg/L implemented over 18 months. Four PacBio metagenomes and 316 metagenome-assembled genomes are announced.

## ANNOUNCEMENT

Reducing dissolved oxygen (DO) levels in biological nutrient removal (BNR) processes can maintain efficient nitrification and phosphorus removal while lowering energy consumption (1–3). Decreased DO causes microbial community adaptation, though the dynamics remain poorly understood (1, 4). We report metagenomes and metagenome-assembled genomes (MAGs) from a BNR pilot plant operated at the pilot testing facility of the Hamptons Road Sanitation District (HRSD) Virginia Initiative Pilot (VIP) treatment plant in Norfolk, VA (5). Grab samples of mixed liquor were collected from the end of the aeration basin of the treatment plant before and after an 18-month transition from high- (2.5 mg/L) to low-DO (0.2 mg/L) conditions. Upon collection, the mixed liquor samples were centrifuged; the cell pellets were frozen, and then shipped overnight to the laboratory.

Genomic DNA was extracted directly from the pellets using the DNeasy PowerSoil Kit (Qiagen, Germantown, MD), then quantified using a Qubit fluorometer (Fisher Scientific, Waltham, MA) and stored at −20°C until sequencing. DNA purity was measured on a NanoDrop One (Fisher Scientific), and concentration was measured with the Qubit dsDNA High-Sensitivity Kit (Fisher Scientific).

**Fig 1:**
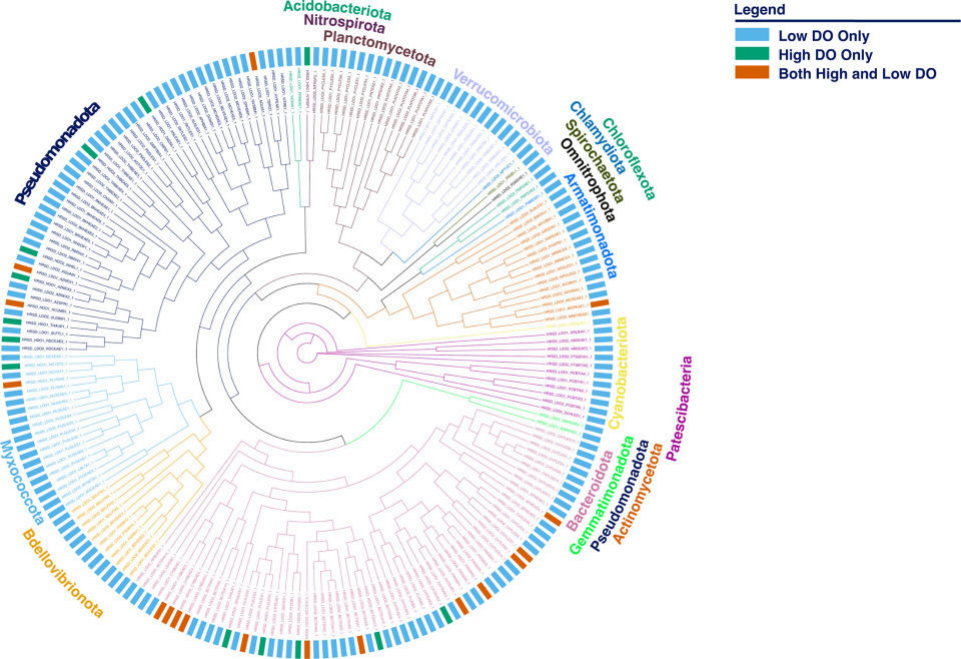
Phylogenetic map of the 207 unique MAG clusters from high and low-DO samples collected from the HRSD pilot plant. The outer ring is color-coded to indicate whether the cluster included MAGs from either low-DO, high-DO, or both conditions. MAG classification is indicated with names matching the color of tree branches.

HiFi library preparation and sequencing at the University of Wisconsin-Madison Biotechnology Center (Madison, WI) followed workflow PN 102-166-600 Version 04 (Pacific Biosciences, Menlo Park, CA) with standard parameters. Library integrity was evaluated on the FemtoPulse System (Agilent, Santa Clara, CA). The library was quantified using the Qubit dsDNA High Sensitivity Kit and sequenced on a Sequel II using Sequel Polymerase Binding Kit 2.2 following the standard protocol (Pacific Biosciences). Across the four samples, there was an average of 1,770,838 reads, with a range from 439,484 to 3,127,946 reads. The average N50 of the reads was 8,664 bp (range of 7,020 bp to 10,234 bp). The Circular Consensus Sequence (CCS) reads were assembled utilizing either metaFLYE (v2.9-b1768) (6) and polished with racon (v1.4.20) (7) for the high-DO samples or metaMDBG (v0.3) (8) with a racon module (v1.4.20) (7) for the low-DO samples. The reads were mapped onto the assemblies using minimap2 (v2.22-r1101) (9). Binning was done with metaBAT2 (v2:2.15) (10). Contaminated contigs within each bin were identified with ProDeGe (v2.3) (11), and custom scripts were used for tetranucleotide frequency analysis (run.GC.sh, Calculating_TF_Correlations.R; https://github.com/GLBRC/metagenome_analysis). Contigs identified as contaminated with both methods were removed from the final MAG assemblies. CheckM (v1.2.2) (12) was used for quality evaluation, and taxonomy was determined using GTDB-Tk (v2.1.0) database release 09-RS214 (13). Functional annotations were assigned using Bakta (v1.9.1) (14). RAxML-NG adaptive (v1.2.1) (15) was used to infer the best phylogenetic tree by maximum likelihood estimation. The tree (Fig. 1) was visualized in TreeViewer (v2.2.0) (16) and annotated in Inkscape (v1.2.2) (https://inkscape.org). We obtained 316 MAGs from four metagenomes, with 278 MAGs from low-DO and 38 from high-DO. These MAGs were dereplicated with dRep (v0.6.1) (17) using a 95% identity cutoff, resulting in 207 unique MAGs with over 75% completeness and less than 10% contamination (Fig. 1 and in the Figshare dataset [18]). As treatment plants begin to embrace the concept of low-DO operation for energy savings (2, 5), these MAGs augment the knowledge of the microbial communities under low-DO operation.

The metagenomes, two from the high-DO and two from the low-DO condition, are available under BioProject number PRJNA1156690. The MAGs and their taxonomy can be accessed on Figshare (10.6084/m9.figshare.30389092 [18]). Custom scripts used in the analysis are available at https://github.com/GLBRC/metagenome_analysis.
